# Coffee Consumption, Genetic Polymorphisms, and the Risk of Type 2 Diabetes Mellitus: A Pooled Analysis of Four Prospective Cohort Studies

**DOI:** 10.3390/ijerph17155379

**Published:** 2020-07-26

**Authors:** An Na Kim, Hyun Jeong Cho, Jiyoung Youn, Taiyue Jin, Moonil Kang, Joohon Sung, Jung Eun Lee

**Affiliations:** 1 Department of Food and Nutrition, Seoul National University, Seoul 08826, Korea; ank1101@snu.ac.kr (A.N.K.); 92hyunjung@snu.ac.kr (H.J.C.); ji0youn@snu.ac.kr (J.Y.); taewol@snu.ac.kr (T.J.); 2 Institute of Health and Environment, Graduate School of Public Health, Seoul National University, Seoul 08826, Korea; kmihoho1@snu.ac.kr; 3 Department of Epidemiology, School of Public Health, Seoul National University, Seoul 08826, Korea; jsung@snu.ac.kr; 4 The Research Institute of Human Ecology, Seoul National University, Seoul 08826, Korea

**Keywords:** coffee, type 2 diabetes mellitus, genetic polymorphism, pooled analysis

## Abstract

The association between coffee consumption and the risk of type 2 diabetes may vary by genetic variants. Our study addresses the question of whether the incidence of type 2 diabetes is related to the consumption of coffee and whether this relationship is modified by polymorphisms related to type 2 diabetes. We performed a pooled analysis of four Korean prospective studies that included 71,527 participants; median follow-up periods ranged between 2 and 13 years. All participants had completed a validated food-frequency questionnaire (FFQ) at baseline. The odds ratios (ORs) and 95% confidence intervals (CIs) for type 2 diabetes were calculated using logistic regression models. The ORs were combined using a fixed or random effects model depending on the heterogeneity across the studies. Compared with 0 to <0.5 cups/day of coffee consumption, the OR for type 2 diabetes was 0.89 (95% CI: 0.80–0.98, *p* for trend = 0.01) for ≥3 cups/day of coffee consumption. We did not observe significant interactions by five single nucleotide polymorphisms (SNPs) related to type 2 diabetes (*CDKAL1* rs7756992, *CDKN2A*/B rs10811661, *KCNJ11* rs5215, *KCNQ1* rs163184, and *PEPD* rs3786897) in the association between coffee and the risk of type 2 diabetes. We found that coffee consumption was inversely associated with the risk of type 2 diabetes.

## 1. Introduction

Diabetes mellitus is a global health problem. The global age-standardized prevalence of diabetes was 8.3% in 2019 and is projected to reach 9.6% by 2045 [1]. Type 2 diabetes is the most prevalent and accounts for 90% of all cases of diabetes mellitus [2]. The prevalence of type 2 diabetes has increased during the last decade within Asian populations [3]. The prevalence of diabetes among Korean adults aged ≥30 years increased from 9.7% to 10.4% between 2008 and 2018 [4]. Type 2 diabetes can be prevented by identifying modifiable risk factors that are imperative for the prevention of type 2 diabetes. The influence of dietary behaviors (healthy dietary patterns, the alternative healthy eating index, and low-carbohydrate diet) and foods (intake of whole grains, red meat, processed meat, and sugar-sweetened beverages) on the incidence of type 2 diabetes has been examined in observational studies and a meta-analysis [5,6,7,8].

Coffee has drawn attention in relation to type 2 diabetes prevention because of its widespread consumption and the effects of polyphenol compounds. Particularly in Korea, along with economic development, coffee consumption is steadily increasing. The average coffee consumption increased to 1.08 cups/day in 2018 from 0.59 cups/day in 2001 [4]. The average frequency of drinking coffee among Korean adults was 11 times/week in 2016, making coffee the most frequently consumed food [9]. Despite the high prevalence of type 2 diabetes and consumption of coffee in Korea, studies on coffee consumption and the risk of type 2 diabetes among Koreans are sparse.

Epidemiological meta-analyses have shown an inverse association between coffee consumption and the risk of type 2 diabetes [10,11,12]. In a recent dose–response meta-analysis, the risk of type 2 diabetes decreased by 6% (relative risk (RR) = 0.94; 95% CI = 0.93–0.95) for each cup/day increase in coffee consumption [13]. Several mechanisms underlying this protective effect of coffee on the risk of type 2 diabetes have been suggested. Biological components in coffee, such as caffeine, chlorogenic acids, lignans, and antioxidants, have been suggested to play a role in regulating insulin and glucose, which influence the process of developing type 2 diabetes [14,15,16].

In addition to lifestyle factors, genetic factors also play a role in the development of type 2 diabetes. Although genome-wide association studies (GWAS) have identified many genetic variant loci related to type 2 diabetes, these variants explain approximately 6% of the susceptibility of type 2 diabetes [17]. It is important to further investigate interaction studies because its interaction with genetic variants may partly explain the remaining susceptibility of type 2 diabetes [18]. Susceptibility to modifiable lifestyle factors depends on genetic factors, which may interact with lifestyle factors [18,19]. Identifying type 2 diabetes risk subgroups based on the type 2 diabetes susceptibility gene, which may be modified by specific foods or nutrients, may help to develop more individualized prevention strategies [19].

A recent trans-ethnic meta-analysis of more than 110,000 individuals, which combined GWAS data of multiple ethnic groups including European, East Asian, South Asian, and Mexican/Mexican African individuals has shown genetic variants related to type 2 diabetes [20]. Among more than 70 genetic variants across the ethnic groups, only four genetic variants have been shown to be significantly (*p* < 1 × 10^−6^) associated with type 2 diabetes in East Asian populations and one genetic variant was significant in both European and East Asian populations [20].

We assumed that the association between coffee consumption and the risk of type 2 diabetes could be modified by type 2 diabetes polymorphisms. Our study addresses the question of whether the incidence of type 2 diabetes is related to the consumption of coffee and whether this relationship varies by type 2 diabetes susceptibility genes. In this study, we prospectively examined the association between coffee consumption and genetic polymorphisms related to type 2 diabetes in four Korean prospective cohort studies.

## 2. Materials and Methods

### 2.1. Study Population

The participants were drawn from the four prospective cohorts of the Korean Genome and Epidemiology study (KoGES): the Health Examinees (HEXA) study, the Cardiovascular Disease Association Study (CAVAS), the Korea Association Resource (KARE) study, and the Healthy Twin (TWIN) study [21]. The HEXA, CAVAS, and KARE studies consisted of community-dwellers and participants recruited from the national health examinee registry who were aged 40 years or older at baseline. The TWIN study consisted of same-sex twins aged 30 years or older at baseline and their first-degree adult family members. The participants were recruited between 2004 and 2013 for the HEXA study, between 2005 and 2011 for the CAVAS, between 2001 and 2002 for the KARE study, and between 2005 and 2013 for the TWIN study.

Follow-up examinations were conducted once from 2012 to 2016 for the HEXA study and from 2007 to 2017 for CAVAS (median follow-up: 4.25 years, 2.08 years, respectively). For the KARE study, a total of six follow-ups were conducted between 2003 and 2016 at 2-year intervals (median follow-up: 11.67 years). For the TWIN study, a total of three follow-ups were conducted from 2008 to 2015 (median follow-up: 3.17 years). Blood samples were drawn at baseline and at each follow-up. Blood samples were collected in a serum separator tube and two ethylenediaminetetraacetic acid (EDTA) tubes. Further information on the study design can be found elsewhere [21]. For the HEXA study and the CAVAS, we included participants who completed the follow-up, and for the KARE and the TWIN studies, we included everyone who participated in at least one follow-up.

For our analysis, there were a total of 214,911 participants at baseline, and we excluded those with the following characteristics: had a history of type 2 diabetes, myocardial infarction, stroke, or cancer at baseline (*n* = 40,386) (*n* = 25,799 for the HEXA study; *n* = 5024 for the CAVAS; *n* = 9196 for the KARE study; *n* = 367 for the TWIN study); did not provide food-frequency questionnaires (FFQs) at baseline (*n* = 3712) (*n* = 3066 for the HEXA study; *n* = 177 for the CAVAS; *n* = 326 for the KARE study; *n* = 143 for the TWIN study); did not provide information on coffee consumption at baseline (*n* = 2318) (*n* = 1905 for the HEXA study; *n* = 144 for the CAVAS; *n* = 246 for the KARE study; *n* = 23 for the TWIN study); had implausible total energy intake at baseline ( ± 3 standard deviations (SDs) from the mean of the log transformed total energy intake) (*n* = 5947) (*n* = 4883 for the HEXA study; *n* = 474 for the CAVAS; *n* = 412 for the KARE study; *n* = 178 for the TWIN study); did not provide blood samples at follow-up (*n* = 134,027) (*n* = 107,725 for the HEXA study; *n* = 15,873 for the CAVAS; *n* = 9257 for the KARE study; *n* = 1172 for the TWIN study); had none of the follow-up information on fasting plasma glucose, HbA1c measurements, self-reported diagnosis of diabetes, and use of diabetes medication (*n* = 2690) (*n* = 6 for the HEXA study; *n* = 3 for the CAVAS; *n* = 2681 for the KARE study; *n* = 0 for the TWIN study). A total of 71,527 participants (*n* = 54,122 for the HEXA study; *n* = 9994 for the CAVAS; *n* = 5698 for the KARE study; *n* = 1713 for the TWIN study) were included in this analysis (Figure S1). In addition, for the subgroup analysis, we further excluded participants who did not have genetic information (*n* = 53,234 for the HEXA study; *n* = 7356 for the CAVAS; *n* = 622 for the KARE study; *n* = 718 for the TWIN study). Therefore, among 71,527 participants, the DNA samples from a total of 9597 participants were genotyped (*n* = 888 for the HEXA study; *n* = 2638 for the CAVAS; *n* = 5076 for the KARE study; *n* = 995 for the TWIN study).

Written informed consent was obtained from all study participants. The study protocol was approved by the Institutional Review Board of Seoul National University (IRB no. E1903/002-002).

### 2.2. Ascertainment of Type 2 Diabetes

The concentrations of fasting plasma glucose and the 2-h plasma glucose levels of the oral glucose tolerance test (OGTT) were measured by the hexokinase method (ADVIA 1650; Bayer, Inc., Tarrytown, NY, USA). In accordance with the type 2 diabetes criteria of the American Diabetes Association, confirmation of type 2 diabetes was defined as the presence of any one of the following [22]: (1) fasting plasma glucose level ≥ 126 mg/dL, (2) HbA1c level ≥ 6.5%, (3) history of diagnosed diabetes, or (4) current use of hypoglycemic medication. Since only the KARE study participants measured the OGTT, an OGTT level ≥ 200 mg/dL was considered confirmation of type 2 diabetes. Incident cases referred to participants who did not have diabetes at baseline and met any of the aforementioned conditions during follow-up—whichever occurred first.

### 2.3. Assessments of Coffee and Other Factors

Coffee and green tea consumption were assessed using a 106-item semi-quantitative FFQ in the HEXA, CAVAS, and TWIN studies and a 103-item semiquantitative FFQ in the KARE study. The validity and reproducibility of the FFQs have been described in detail elsewhere [23,24]. Participants were asked to select from nine categories of coffee consumption frequency over the preceding year, ranging from almost never to 5 or more times per day in the HEXA, CAVAS, and TWIN studies and ranging from almost never to 3 or more times per day in the KARE study. The frequency of the use of coffee additives such as cream and sugar was also collected. The portion size was composed of three categories (a half of, equal to, and 2 times a standard serving size for HEXA, CAVAS, KARE, and TWIN studies). To estimate the pooled odds ratios (ORs) of the four prospective cohort studies, the categories of coffee consumption were unified into four categories: 0 to <0.5, 0.5 to <1, 1 to <3, and ≥3 cups per day. Green tea consumption was also assessed using the FFQs in the same way that coffee consumption was calculated.

Trained interviewers obtained information on the following demographic and lifestyle factors: education level; smoking status (never, past, and current smoker), the number of years spent smoking, and the number of cigarettes smoked daily; alcohol drinking status (never, past, and current drinker), frequency of alcohol drinking, and serving size of alcohol across the studies. Physical activity was also assessed (none, frequency 1–2, 3–4, 5–6, and every day per week for the HEXA, CAVAS, TWIN studies). For the KARE study, the frequency and duration of types of physical activity (aerobics, jogging, swimming, tennis, golf, bowling, walking, and climbing) were assessed.

Pack-years of smoking were calculated by dividing the daily number of cigarettes by 20 and multiplying this result by the number of years spent smoking. Total alcohol consumption is calculated as grams of ethanol per day [25]. Body mass index (BMI) was calculated as the weight in kilograms divided by the square of the height in meters. Metabolic equivalent hours (MET-h)/week was determined by multiplying the hours per week spent in each activity by the metabolic cost of each activity in METs [26].

### 2.4. Genotyping and Single Nucleotide Polymorphisms (SNPs) Selection

The genomic DNA samples isolated from the peripheral blood of the study participants in the HEXA and TWIN studies were directly genotyped with the Affymetrix Genome-Wide Human SNP array 6.0 [27]. Individual genotypes were called with the birdseed genotyping algorithm [27]. Some of the CAVAS participants were genotyped by the aforementioned method and the rest of the CAVAS participants were genotyped using the Illumina Omni 1 Quad bead microarrays, isolated from peripheral blood drawn from the CAVAS participants [27,28]. Participants in the KARE study were genotyped with the Affymetrix Genome-Wide Human SNP Array 5.0 [29]. The individual genotypes were called by applying the Bayesian robust linear model using the Mahalanobis distance genotyping algorithm in the KARE study [29]. We further imputed the non-typed or missing genotypes for each participant in the KARE study, using IMPUTE v246 with 1000 Genomes data [27]. Genotyping with Affymetrix 5.0, Affymetrix 6.0, and Illumina Omni 1 M and quality control procedures have been described in detail previously [27,28,29].

In our study, we extracted 5 single nucleotide polymorphisms (SNPs) related to type 2 diabetes (cyclin-dependent kinase 5 regulatory subunit-associated protein 1-like 1 (*CDKAL1*) rs7756992, cyclin-dependent kinase inhibitor-2A/B (*CDKN2A/B*) rs10811661, potassium inwardly rectifying channel subfamily J member 11 (*KCNJ11*) rs5215, potassium voltage-gated channel KQT-like subfamily member 1 (*KCNQ1*) rs163184, and peptidase D (*PEPD*) rs3786897) with suggestive significance (*p* < 1 × 10^−6^) among East Asian populations based on evidence from a trans-ethnic meta-analysis [20]. Four of the aforementioned 5 SNPs (*CDKAL1* rs7756992, *CDKN2A/B* rs10811661, *KCNJ11* rs5215, *KCNQ1* rs163184) were all available from direct genotyping within the HEXA, CAVAS, KARE, and TWIN studies. Information on the remaining SNP (*PEPD* rs3786897) was only available within the CAVAS and the KARE studies.

### 2.5. Statistical Analysis

We used multivariate logistic regression models to examine the associations between coffee consumption and the risk of type 2 diabetes. We used logistic regression to calculate the odds ratios (ORs) and 95% confidence intervals (CIs) because we included participants who provided blood samples at both follow-up and baseline and ascertained type 2 diabetes by fasting plasma glucose level ≥ 126 mg/dL, HbA1c level 6.5%, or OGTT level ≥ 200 mg/dL. We estimated ORs and 95% CIs according to the categories of coffee consumption. The chi-square test was used to compare categorical variables and the ANOVA test was used to compare continuous variables. In multivariable analyses, we adjusted for age (years, continuous), sex, BMI (<18.5, 18.5 to <23, 23 to <25, and ≥25 kg/m^2^), alcohol intake (never, ethanol g/day < 10, 10–<20, 20–<30, 30–<40, 40–<50, 50–<60, ≥60 for men and never, ethanol g/d <10, 10–<20, ≥20 for women for HEXA and KARE; never, ethanol g/day < 10, 10–<20, 20–<30, 30–<40, ≥40 for men and never, ethanol g/d < 10, ≥10 for women for CAVAS; never, ethanol g/day < 10, ≥10 for men and never, ever for women for TWIN), smoking status (never, pack-years < 10, 10–<20, 20–<30, ≥30 for men and never, pack-years < 5, 5–<10, ≥10 for women for HEXA; never, pack-years < 10, 10–<20, 20–<30, ≥30 for men and never, pack-years < 5, ≥5 for women for CAVAS; never, pack-years < 10, 10–<20, 20–<30, 30–<40, ≥40 for men and never, pack-years < 5, 5–<10, ≥10 for women for KARE; never/ever for men and never/ever for women for TWIN), regular exercise (none, physical activity frequency 1–2, 3–4, 5–6, and every day per week in HEXA, CAVAS, TWIN/ tertile in MET-h/week in KARE), education level (elementary school or less, middle school, and high school or above), green tea intake (0 to <1, 1 to <2, and ≥2 cups/day), and total energy intake (kcal/day, continuous). Since adjustment for fruit and vegetable intake, meat intake, and dairy intake did not change our estimates appreciably, we did not include them in our final model. To test for linear trends across categories of coffee consumption, we modeled the median of each category of coffee consumption as a continuous variable. The pooled ORs were estimated using a random effects model when there was evidence of heterogeneity or a fixed effect model when there was no heterogeneity [30]. We tested heterogeneity across the studies using the Q statistics [31].

We additionally performed subgroup analyses to examine whether the associations between coffee consumption and type 2 diabetes varied by age (age <50 or ≥50 years), sex, BMI (<25 or ≥25 kg/m^2^), smoking status (never smoker or ever smoker), alcohol drinking (nondrinker or current drinker), and type 2 diabetes susceptibility genes using a meta-regression model [32]. The test for nonlinearity of the association was performed using restricted cubic splines [33]. For the cubic spline analysis, all studies were combined into a single dataset and then adjusted for covariates. The top 1% of participants in the aggregated dataset were excluded from the cubic spline analysis to reduce the excessive influence of extreme coffee consumption. All statistical tests were two-sided, and *p* values less than 0.05 were considered statistically significant. All analyses were performed using SAS software, version 9.4 (SAS Institute Inc, Cary, NC, USA), and STATA SE 15 (Stata Corporation, College Station, TX, USA) for the meta-regression.

## 3. Results

### 3.1. Baseline Characteristics of Participants

A total of 4600 incident type 2 diabetes cases were identified among participants during median follow-up periods of 2–13 years across the four studies. The baseline characteristics of the study population according to the consumption of coffee are presented in Table 1. At baseline, participants with higher coffee consumption were of younger age, had higher levels of smoking and alcohol drinking, and had higher education levels. Coffee drinkers were more likely to have higher energy intake and consume green tea than nondrinkers.

### 3.2. Association Between Coffee Consumption and the Risk of Type 2 Diabetes

**Figure 1:**
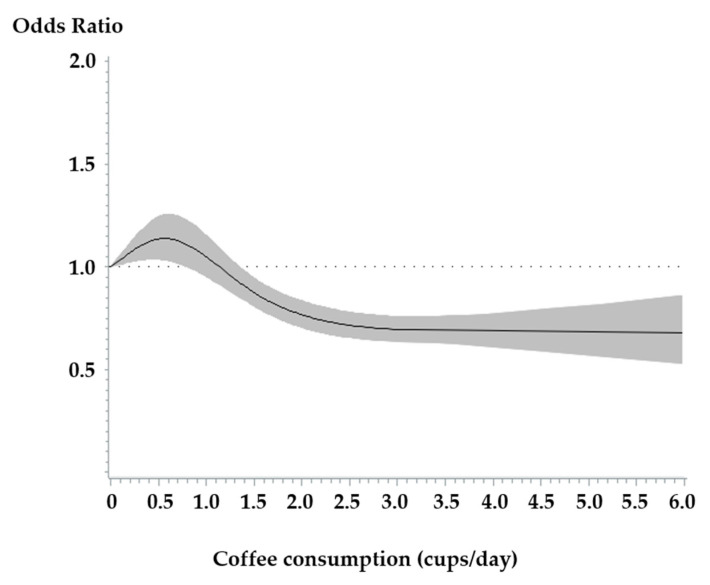
Continuous dose–response association between coffee consumption and the risk of type 2 diabetes with restricted cubic splines. The solid line represents the estimated odds ratios (ORs), and the shaded area represents 95% confidence intervals (CIs). *p* for curvature = 0.0001.

We found an inverse association between coffee consumption and risk of type 2 diabetes. Compared to 0 to <0.5 cups/day of coffee consumption, pooled ORs (95% CIs) were 1.02 (0.91–1.14) for 0.5 to <1 cups/day of coffee consumption, 0.97 (0.90–1.05) for 1 to <3 cups/day of coffee consumption, and 0.89 (0.80–0.98) for ≥3 cups/day of coffee consumption (*p* for trend = 0.01) (Table 2). The association was inverse in each cohort: compared 0 to <0.5 cups/day of coffee consumption, ORs (95% CIs) were 0.90 (0.80–1.01; *p* for trend = 0.04) for ≥3 cups/day of coffee consumption in the HEXA study, 0.84 (0.57–1.24; *p* for trend = 0.38) for ≥3 cups/day of coffee consumption in the CAVAS, 0.88 (0.71–1.09; *p* for trend = 0.29) for ≥3 cups/day of coffee consumption in the KARE study, and 0.82 (0.33–1.06; *p* for trend = 0.65) for ≥3 cups/day of coffee consumption in the TWIN study. In a restricted cubic spline analysis, we found that coffee consumption was nonlinearly associated with a lower risk of type 2 diabetes (*p* for curvature = 0.0001, Figure 1). We observed a decrease in the risk of type 2 diabetes when 0.6 cups/day to 3 cups/day of coffee were consumed, but we did not observe any apparent further decrease in the risk of type 2 diabetes above ≥3 cups/day.

### 3.3. Subgroup Analyses for the Association Between Coffee Consumption and Type 2 Diabetes

The inverse association between coffee consumption and the risk of type 2 diabetes persisted in subgroup analyses according to age, sex, BMI, smoking status, and alcohol intake (*p* for interaction = 0.24, 0.51, 0.12, 0.31, and 0.73, respectively) (Table 3). We also examined whether the association between coffee consumption and the risk of type 2 diabetes was modified by type 2 diabetes susceptibility genes. The inverse association did not vary by polymorphisms of rs7756992 in *CDKAL1*, rs10811661 in *CDKN2A/B*, rs5215 in *KCNJ11*, rs163184 in *KCNQ1*, and rs3786897 in *PEPD* (*p* for interaction = 0.56, 0.97, 0.73, 0.62, and 0.68, respectively) (Table 4). In the recessive model, the inverse association did not vary by polymorphisms of rs7756992 in *CDKAL1*, rs10811661 in *CDKN2A/B*, rs5215 in *KCNJ11*, rs163184 in *KCNQ1*, and rs3786897 in *PEPD* (*p* for interaction = 0.18, 0.31, 0.60, 0.80, 0.80, respectively) (Table S1). In the additive model, the inverse association did not vary by polymorphisms of rs7756992 in *CDKAL1*, rs10811661 in *CDKN2A/B*, rs5215 in *KCNJ11*, rs163184 in *KCNQ1*, and rs3786897 in *PEPD* (*p* for interaction = 0.66, 0.46, 0.73, 0.66, 0.52, respectively) (Table S2).

## 4. Discussion

In this pooled analysis of 71,527 participants from four Korean prospective studies, we found that the consumption of ≥3 cups/day of coffee compared to 0 to <0.5 cups/day of coffee was associated with an 11% lower statistically significant risk of type 2 diabetes. The association between coffee consumption and the risk of incidence of type 2 diabetes did not vary by type 2 diabetes susceptibility genes.

Our results are consistent with the inverse association between coffee consumption and the risk of type 2 diabetes shown by other observational studies and meta-analyses performed in diverse countries [13,34,35]. In a recent systematic review and meta-analysis, the pooled RR was 0.71 (95% CI = 0.67–0.76) in the highest category compared to the lowest category [13].

We found no evidence of interaction with coffee consumption and risk of type 2 diabetes for *CDKAL1* rs7756992, *CDKN2A/B* rs10811661, *KCNJ11* rs5215, *KCNQ1* rs163184, and *PEPD* rs3786897. In line with our results, a previous study failed to find any interaction between rs163184 in *KCNQ1* and coffee consumption in relation to type 2 diabetes [36]. We observed no interaction with rs7756992 in *CDKAL1* (*p* for interaction = 0.56). However, our previous study found an interaction between the risk-conferring G-allele of *CDKAL1* variants in coffee consumption and type 2 diabetes and prediabetes [37], but, when we further expanded our analysis to a larger population, we did not observe a significant interaction anymore. Similarly, previous interaction findings for type 2 diabetes susceptibility genes, such as *TCF7L2*, *GIPR*, *CAV2*, and *HFE*, with other dietary factors were not replicated in the EPIC-InterACT study [38]. One possible reason for this discrepancy may be due to an inappropriate sample size that resulted in insufficiently powered statistical analysis [39].

Genetic polymorphisms in *CDKAL1*, *CDKN2A/2B*, *KCNJ11*, *KCNQ1*, and *PEPD* that we selected as potential effect modifiers were found to be associated with type 2 diabetes in the East Asian population [20]. *CDKAL1* and *CDKN2A/2B* have been associated with reduced insulin secretion by decreasing pancreatic β-cell function [40]. *CDKAL1* shares considerable domain with CDK5 regulator subunit-associated protein (CDK5 Rap1) [41,42]. Since CDK5 is implicated in insulin secretion in pancreatic β-cells, *CDKAL1* may regulate insulin secretion through interaction with CDK5 [41,43]. *PEPD* attenuated insulin-induced AKT2 phosphorylation, resulting in increased insulin resistance [44]. The potassium ion channels are responsible for the first phase if insulin secretion does not respond to glucose. The potassium channel gene *KCNJ11* changed promoter methylation states in diabetic islets which impaired insulin secretion [45]. *KCNQ1* is expressed in tissues including the brain, adipose tissue, pancreas, and the insulin-secreting cell line INS1 [46]. The risk allele of *KCNQ1* for type 2 diabetes is also associated with impaired insulin secretion, suggesting that the risk allele might confer susceptibility [47]. Thus, the risk allele may influence the development of type 2 diabetes through the increased expression of *KCNQ1* in pancreatic β cells [47].

Coffee consumption has been associated with improved insulin sensitivity [14,15,16]. Given the many bioactive components in coffee, several mechanisms have suggested that coffee consumption might reduce the risk of type 2 diabetes [14,15,16]. Chlorogenic acids are the major phenolic components in coffee and their hypoglycemic effects may be inhibiting glucose absorption and stimulating insulin secretion [16]. Chlorogenic acid may suppress glucose release from the liver and improve glucose uptake [48]. In animal studies, consumption of chlorogenic acid has been shown to reduce fasting plasma glucose and increase insulin sensitivity [49].

In addition, coffee contains several antioxidants, such as caffeine, chlorogenic acid, lignan, and melanoidins, which may suppress oxidative stress, thus contributing to better metabolic control [50,51,52]. Acute intake and long-term coffee consumption have been demonstrated to lower oxidative stress in both human and animal studies [53,54]. Several components of coffee have been shown to have anti-inflammatory properties, such as caffeine, chlorogenic acid, cafestrol, trigonelline, and kahweol [14,55]. Epidemiological and clinical studies have shown that coffee consumption may reduce the levels of pro-inflammatory biomarkers such as interleukin(IL)-1b, IL-4, IL-6, IL-10, and C-reactive proteins, which can contribute to a reduced risk of type 2 diabetes [55,56,57].

The current pooled analysis had several limitations. Firstly, measurement errors inherent in the dietary assessment may be present. However, several epidemiological studies have reported that coffee consumption by the FFQ is well measured, with reasonable validity [58,59]. Secondly, the type of coffee consumed (caffeinated, decaffeinated) or brewing methods (unfiltered coffee or filtered coffee) were not assessed in the studies. However, there was an implication of consistent results despite the different coffee types (caffeinated or decaffeinated) [60,61,62] or brewing methods (unfiltered coffee or filtered coffee) [63,64]. Thirdly, we had limited ability to examine the association between coffee and incidence of type 2 diabetes at high levels of coffee consumption. Although we were able to categorize coffee consumption up to 6 or 10 cups/day, we used ≥3 cups/day as the highest level of coffee consumption because only a few participants reached up to 10 cups/day. This result may reflect the actual patterns of coffee consumption among Koreans. Fourthly, as an observational study, the potential for residual confounding could not be ruled out. However, we carefully adjusted for possible confounding factors, including smoking and alcohol intake. Fifthly, the high rate of failure to revisit the clinic for a health examination may be a concern. For the KARE study, approximately 90% of baseline participants completed at least one follow-up survey, but the rate of revisits to the clinic for a health examination decreased to 60% by the sixth follow-up [21]. For the HEXA, CAVAS, and TWIN studies, the rate of revisits to the clinic for a health examination decreased by approximately 40%–60%. Therefore, our results may not be representative of the full cohort.

The current analysis has several strengths. Firstly, we were able to increase statistical power by combining the results of four individual studies. Otherwise, the number of cases available for examination was insufficient to analyze the association of each cohort study. Secondly, we were able to model potential confounding variables consistently and adjust for greater specificity across the studies to attenuate any potential heterogeneity in the results. Thirdly, we had a large sample size (*n* = 71,527) of the Asian population.

## 5. Conclusions

In conclusion, coffee consumption was significantly associated with a reduced risk of type 2 diabetes incidence. We did not detect statistically significant interactions between coffee consumption and five SNPs related to type 2 diabetes (*CDKAL1* rs7756992, *CDKN2A/B* rs10811661, *KCNJ11* rs5215, *KCNQ1* rs163184, and *PEPD* rs3786897) in the association between coffee and the risk of type 2 diabetes. Further replication of gene and diet interactions for type 2 diabetes in Asian populations is needed.

## Figures and Tables

**Table 1:** Baseline characteristics of the study population according to categories of coffee consumption.

	Coffee Consumption (cups/Day)				
Study	0 to <0.5	0.5 to <1	1 to <3	≥3	*p* Value ^2^
**HEXA**					
Total population no.	15,480	5310	22,598	10,734	
Age at baseline (years of age)	54.51 ± 7.82	52.73 ± 7.86	52.89 ± 7.87	51.12 ± 7.70	<0.001
Sex					<0.001
Men	3980 (25.71)	1692 (31.86)	6883 (30.46)	4981 (46.40)	
Women	11,500 (74.29)	3618 (68.14)	15,715 (69.54)	5753 (53.60)	
BMI (kg/m^2^)					<0.001
<18.5	354 (2.29)	88 (1.66)	344 (1.52)	147 (1.37)	
18.5–<23	6848 (44.27)	2082 (39.23)	8689 (38.46)	3839 (35.79)	
23–<25	4266 (27.58)	1546 (29.13)	6433 (28.47)	3087 (28.78)	
≥25	4001 (25.86)	1591 (29.98)	7126 (31.54)	3654 (34.06)	
Cigarette smoking (pack-years) ^1^	2.79 ± 8.57	4.13 ± 10.41	4.47 ± 10.84	9.33 ± 15.35	<0.001
Alcohol drinking (g/day) ^1^	4.98 ± 26.92	6.00 ± 16.80	6.03 ± 15.20	9.19 ± 21.81	<0.001
Regular exercise ^1^					<0.001
no	6572 (42.61)	2151 (40.59)	10,069 (44.72)	5279 (49.30)	
yes	8852 (57.39)	3148 (59.41)	12,447 (55.28)	5428 (50.70)	
Education level ^1^					<0.001
Elementary school or less	2778 (18.20)	643 (12.22)	2996 (13.42)	1026 (9.65)	
Middle school	2828 (18.53)	754 (14.33)	3462 (15.51)	1443 (13.57)	
High school or above	9655 (63.27)	3863 (73.44)	15,866 (71.07)	8163 (76.78)	
Total energy intake (kcal/day)	1649.55 ± 493.61	1697.68 ± 494.80	1780.62 ± 490.32	1881.3 ± 541.84	<0.001
Green tea consumption (cups/day)					<0.001
0–<1	6057 (39.13)	1583 (29.81)	8864 (39.22)	4727 (44.04)	
1–<2	7653 (49.44)	3134 (59.02)	9211 (40.76)	3919 (36.51)	
≥2	1770 (11.44)	593 (11.17)	4523 (20.02)	2088 (19.45)	
**CAVAS**					
Total population no.	3375	889	3991	1739	
Age at baseline (years of age)	61.84 ± 9.07	59.79 ± 9.25	60.50 ± 9.09	58.50 ± 9.27	<0.001
Sex					<0.001
Men	1038 (30.76)	354 (39.82)	1451 (36.36)	937 (53.88)	
Women	2337 (69.24)	535 (60.18)	2540 (63.64)	802 (46.12)	
BMI (kg/m^2^)					<0.001
<18.5	70 (2.07)	17 (1.91)	64 (1.60)	30 (1.73)	
18.5-<23	1238 (36.68)	278 (31.27)	1198 (30.02)	549 (31.57)	
23-<25	865 (25.63)	218 (24.52)	1061 (26.58)	445 (25.59)	
≥25	1202 (35.61)	376 (42.29)	1668 (41.79)	715 (41.12)	
Cigarette smoking (pack-years) ^1^	4.75 ± 13.26	6.99 ± 15.64	7.15 ± 15.96	14.17 ± 21.63	<0.001
Alcohol drinking (g/day) ^1^	7.01 ± 22.73	10.69 ± 25.78	8.40 ± 22.47	10.26 ± 23.08	<0.001
Regular exercise ^1^					0.141
no	2303 (68.34)	580 (65.24)	2756 (69.11)	1174 (67.55)	
yes	1067 (31.66)	309 (34.76)	1232 (30.89)	564 (32.45)	
Education level ^1^					<0.001
Elementary school or less	2085 (61.98)	433 (48.82)	2165 (54.30)	754 (43.43)	
Middle school	532 (15.81)	150 (16.91)	713 (17.88)	345 (19.87)	
High school or above	747 (22.21)	304 (34.27)	1109 (27.82)	637 (36.69)	
Total energy intake (kcal/day)	1541.67 ± 474.21	1634.86 ± 492.65	1679.10 ± 469.95	1846.12 ± 545.15	<0.001
Green tea consumption (cups/day)					<0.001
0–<1	1351 (40.03)	346 (38.92)	1826 (45.75)	825 (47.44)	
1–<2	1676 (49.66)	478 (53.77)	1479 (37.06)	592 (34.04)	
≥2	348 (10.31)	65 (7.31)	686 (17.19)	322 (18.52)	
**KARE**					
Total population no.	2079	546	2280	793	
Age at baseline (years of age)	53.41 ± 8.70	50.71 ± 8.16	50.20 ± 8.14	48.60 ± 7.48	<0.001
Sex					<0.001
Men	804 (38.67)	280 (51.28)	1027 (45.04)	551 (69.48)	
Women	1275 (61.33)	266 (48.72)	1253 (54.96)	242 (30.52)	
BMI (kg/m^2^)					<0.001
<18.5	42 (2.02)	3 (0.55)	25 (1.10)	14 (1.77)	
18.5–<23	696 (33.48)	148 (27.11)	659 (28.90)	214 (26.99)	
23–<25	524 (25.20)	159 (29.12)	622 (27.28)	209 (26.36)	
≥25	817 (39.30)	236 (43.22)	974 (42.72)	356 (44.89)	
Cigarette smoking (pack-years) ^1^	5.61 ± 12.29	8.19 ± 13.33	8.37 ± 14.72	17.35 ± 19.62	<0.001
Alcohol drinking (g/day) ^1^	6.86 ± 17.69	9.64 ± 19.92	9.35 ± 21.12	13.22 ± 25.44	<0.001
Exercise (MET-h/wk) ^1^	1596.83 ± 970.33	1574.97 ± 985.81	1507.31 ± 911.05	1441.54 ± 961.17	<0.001
Education level ^1^					<0.001
Elementary school or less	1315 (63.68)	274 (50.46)	1108 (48.85)	305 (38.56)	
Middle school	538 (26.05)	182 (33.52)	810 (35.71)	329 (41.59)	
High school or above	212 (10.27)	87 (16.02)	350 (15.43)	157 (19.85)	
Total energy intake (kcal/day)	1861.91 ± 614.64	1933.38 ± 609.98	1971.82 ± 589.86	2115.02 ± 647.64	<0.001
Green tea consumption (cups/day)					<0.001
0–<1	795 (38.24)	170 (31.14)	857 (37.59)	361 (45.52)	
1–<2	1063 (51.13)	330 (60.44)	982 (43.07)	281 (35.44)	
≥2	221 (7.89)	46 (8.42)	441 (19.34)	151 (19.05)	
**TWIN**					
Total population no.	478	252	590	393	
Age at baseline (years of age)	43.21 ± 14.66	43.99 ± 13.78	42.58 ± 11.60	41.73 ± 10.12	0.121
Sex					<0.001
Men	161 (33.68)	86 (34.13)	209 (35.42)	194 (49.36)	
Women	317 (66.32)	166 (65.87)	381 (64.58)	199 (50.64)	
BMI (kg/m^2^)					0.012
<18.5	18 (3.77)	11 (4.37)	6 (1.02)	10 (2.54)	
18.5–<23	226 (47.38)	104 (41.27)	265 (44.92)	154 (39.19)	
23–<25	110 (23.06)	57 (22.62)	145 (24.58)	93 (23.66)	
≥25	123 (25.79)	80 (31.75)	174 (29.49)	136 (34.61)	
Cigarette smoking (pack-years) ^1^	2.54 ± 7.75	3.22 ± 8.24	4.37 ± 9.57	9.55 ± 16.96	<0.001
Alcohol drinking (g/day) ^1^	7.09 ± 16.94	7.36 ± 14.26	8.94 ± 18.36	16.36 ± 53.90	<0.001
Regular exercise ^1^					0.176
no	291 (62.58)	161 (65.18)	384 (66.67)	270 (70.13)	
yes	174 (37.42)	86 (34.82)	192 (33.33)	115 (29.87)	
Education level ^1^					0.051
Elementary school or less	75 (15.82)	30 (11.95)	64 (10.87)	38 (9.69)	
Middle school	28 (5.91)	24 (9.56)	38 (6.45)	28 (7.14)	
High school or above	371 (78.27)	197 (78.49)	487 (82.68)	326 (83.16)	
Total energy intake (kcal/day)	1815.86 ± 707.23	1869.54 ± 647.56	1904.06 ± 632.84	2069.78 ± 697.32	<0.001
Green tea consumption (cups/day)					<0.001
0–<1	142 (29.71)	49 (19.44)	147 (24.92)	100 (25.45)	
1–<2	210 (43.93)	125 (49.60)	214 (36.27)	132 (33.59)	
≥2	126 (26.36)	88 (30.96)	229 (38.81)	161 (40.96)	

Abbreviations: HEXA, Health Examinee; CAVAS, Cardiovascular Disease Association Study; KARE, the Korea Association Resource; TWIN, the Healthy Twin study; BMI, Body Mass Index; MET-h/wk, metabolic equivalent hours. ^1^ The total number of participants (n = 54,122 for the HEXA study; n = 9994 for the CAVAS; n = 5698 for the KARE study; n = 1713 for the TWIN study) were different in each of the studies because of missing values. ^2^ *p*-value was evaluated by ANOVA for continuous variables and by the chi-square test for categorical variables *p* < 0.05. Continuous variables are reported as mean ± standard deviation and categorical variables are reported as no. (%).

**Table 2:** Odds ratios (ORs) and 95% confidence intervals (CIs) for the risk of type 2 diabetes according to coffee consumption.

	Coffee Consumption (cups/Day)					*p* for Trend	*p* for Heterogeneity ^1^
Study	Median Follow-Up Period (Years)	0 to <0.5	0.5 to <1	1 to <3	≥3		
HEXA	4.25						
Case/Total no.		810/15,480	299/5310	1199/22,598	561/10,734		
Age-sex adjusted OR (CIs)		1.00 (reference)	1.12 (0.97–1.28)	1.05 (0.96–1.15)	1.03 (0.92–1.15)	0.94	
MV adjusted OR (CIs)		1.00 (reference)	1.05 (0.92–1.21)	0.97 (0.88–1.06)	0.90 (0.80–1.01)	0.04	
CAVAS	2.08						
Case/Total no.		92/3375	28/889	121/3991	43/1739		
Age-sex adjusted OR (CIs)		1.00 (reference)	1.17 (0.76–1.80)	1.12 (0.85–1.48)	0.90 (0.62–1.31)	0.62	
MV adjusted OR (CIs)		1.00 (reference)	1.11 (0.72–1.71)	1.05 (0.79–1.39)	0.84 (0.57–1.24)	0.38	
KARE	11.67						
Case/Total no.		515/1564	126/420	556/1724	192/601		
Age-sex adjusted OR (CIs)		1.00 (reference)	0.95 (0.76–1.19)	1.06 (0.92–1.22)	1.02 (0.84–1.25)	0.71	
MV adjusted OR (CIs)		1.00 (reference)	0.90 (0.72–1.14)	0.99 (0.85–1.15)	0.88 (0.71–1.09)	0.29	
TWIN	3.17						
Case/Total no.		18/478	10/252	17/590	13/393		
Age-sex adjusted OR (CIs)		1.00 (reference)	1.04 (0.44–1.46)	0.86 (0.43–1.68)	1.08 (0.48–1.40)	0.90	
MV adjusted OR (CIs)		1.00 (reference)	1.00 (0.42–1.39)	0.75 (0.38–1.48)	0.82 (0.33–1.06)	0.65	
Pooled							
MV adjusted OR (CIs)		1.00 (reference)	1.02 (0.91–1.14)	0.97 (0.90–1.05)	0.89 (0.80–0.98)	0.01	0.99

Abbreviations: MV, multivariate; ORs, odds ratios; CIs, confidence intervals; HEXA, Health Examinee; CAVAS, Cardiovascular Disease Association Study; KARE, the Korea Association Resource; TWIN, the Healthy Twin study. MV adjusted: age (years, continuous), sex (men, women), BMI (<18.5, 18.5–<23, 23–<25, and ≥25 kg/m^2^), alcohol intake (never, ethanol g/day < 10, 10–<20, 20–<30, 30–<40, 40–<50, 50–<60, ≥60 for men; never, ethanol g/d < 10, 10–<20, ≥20 for women for HEXA and KARE, never, ethanol g/day < 10, 10–<20, 20–<30, 30–<40, ≥40 for men; never, ethanol g/d <10, ≥10 for women for CAVAS, and never, ethanol g/day < 10, ≥10 for men; never, ever for women for TWIN), smoking status (never, pack-years < 10, 10–<0, 20–<30, ≥30 for men; never, pack-years < 5, 5–<10, ≥10 for women for HEXA, never, pack-years < 10, 10–<20, 20–<30, ≥30 for men; never, pack-years < 5, ≥ 5 for women for CAVAS, never, pack-years < 10, 10–<20, 20–<30, 30–<40, ≥40 for men; never, pack-years < 5, 5–<10, ≥10 for women for KARE, never/ever for men and never/ever for women for TWIN), regular exercise (no, physical activity frequency 1–2, 3–4, 5–6, and every day per week for HEXA, CAVAS, TWIN, and tertile in MET-h/wk for KARE), education level (elementary school or less, middle school, and high school or above), green tea intake (0–<1, 1–<2, and ≥2 cups/day), and total energy intake (kcal/day, continuous). ^1^ *p* for heterogeneity of odds ratios for top vs. bottom categories in MV adjusted model was presented.

**Table 3:** Subgroup analysis of the association of coffee consumption with type 2 diabetes.

		Coffee Consumption (cups/Day)				*p* for Trend	*p* for Interaction	*p* for Heterogeneity ^1^
Subgroup		0 to <0.5	0.5 to <1	1 to <3	≥3			
Age at baseline (years)							0.24	
<50	Case/Total no.	355/5811	131/2522	559/10,335	311/5986			
	Pooled OR (CIs)	1.00 (reference)	0.84 (0.68–1.05)	0.87 (0.75–1.01	0.79 (0.67–0.95)	0.01		0.49
≥50	Case/Total no.	1080/15,601	332/4475	1334/19,124	498/7673			
	Pooled OR (CIs)	1.00 (reference)	1.10 (0.96–1.26)	1.01 (0.93–1.11)	0.91 (0.81–1.03)	0.11		0.63
Sex							0.51	
Men	Case/Total no.	527/5983	216/2412	850/9570	503/6663			
	Pooled OR (CIs)	1.00 (reference)	1.03 (0.86–1.23)	1.01 (0.89–1.14)	0.86 (0.75–0.99)	0.02		0.97
Women	Case/Total no.	908/15,429	247/4585	1043/19,889	306/6996			
	Pooled OR (CIs)	1.00 (reference)	1.02 (0.88–1.19)	0.95 (0.86–1.04)	0.93 (0.80–1.07)	0.15		0.86
BMI (kg/m^2^)							0.12	
<25	Case/Total no.	766/15,257	211/4711	846/19,511	335/8791			
	Pooled OR (CIs)	1.00 (reference)	0.96 (0.82–1.14)	0.90 (0.81–1.00)	0.80 (0.69–0.92)	<0.01		0.34
≥25	Case/Total no.	669/6143	252/2283	1046/9942	473/4861			
	Pooled OR (CIs)	1.00 (reference)	1.09 (0.93–1.28)	1.04 (0.93–1.16)	0.96 (0.83–1.10)	0.27		0.45
Smoking status							0.31	
Never	Case/Total no.	1011/17,420	290/5233	1197/21,918	341/7769			
	Pooled OR (CIs)	1.00 (reference)	1.06 (0.96–1.17)	1.01 (0.94–1.07)	0.93 (0.84–1.02)	0.18		0.47
Ever	Case/Total no.	411/3894	169/1741	689/7427	466/5852			
	Pooled OR (CIs)	1.00 (reference)	1.06 (0.92–1.21)	0.99 (0.91–1.08)	0.89 (0.81–0.98)	0.01		0.81
Alcohol drinking							0.73	
Non-drinker	Case/Total no.	889/14,070	200/3687	944/15,277	323/5993			
	Pooled OR (CIs)	1.00 (reference)	0.92 (0.78–1.09)	1.00 (0.90–1.11)	0.87 (0.75–1.00)	0.11		0.87
Current drinker	Case/Total no.	540/7251	260/3282	941/14,070	483/7617			
	Pooled OR (CIs)	1.00 (reference)	1.13 (0.96–1.33))	0.95 (0.85- 1.07)	0.90 (0.78- 1.04)	0.04		0.70

Abbreviations: ORs, odds ratios; CIs, confidence intervals; BMI, body mass index. MV adjusted: age (years, continuous), sex (men, women), BMI (<18.5, 18.5–<23, 23–<25, and ≥25 kg/m^2^), alcohol intake (never, ethanol g/day < 10, 10–<20, 20–<30, 30–<40, 40–<50, 50–<60, ≥60 for men; never, ethanol g/d < 10, 10<20, ≥20 for women for HEXA and KARE, never, ethanol g/day < 10, 10–<20, 20–<30, 30–<40, ≥40 for men; never, ethanol g/d < 10, ≥10 for women for CAVAS, and never, ethanol g/day < 10, ≥10 for men; never, ever for women for TWIN), smoking status (never, pack-years < 10, 10–<20, 20–<30, ≥30 for men; never, pack-years < 5, 5–<10, ≥10 for women for HEXA, never, pack-years < 10, 10–<20, 20–<30, ≥30 for men; never, pack-years < 5, ≥5 for women for CAVAS, never, pack-years < 10, 10–<20, 20–<30, 30–<40 ≥40 for men; never, pack-years < 5, 5–<10, ≥10 for women for KARE, never, ever for men and never, ever for women for TWIN), regular exercise (no, physical activity frequency 1–2, 3–4, 5–6, and every day per week for HEXA, CAVAS, TWIN, and tertile category in MET-h/wk for KARE), education level (elementary school or less, middle school, and high school or above), green tea intake (0–<1, 1–<2, and ≥2 cups/day), and total energy intake (kcal/day, continuous). ^1^ *p* for heterogeneity of odds ratios for top vs. bottom categories in MV adjusted model was presented.

**Table 4:** Subgroup analysis on the association of coffee consumption with type 2 diabetes stratified by type 2 diabetes-related SNPs.

Subgroup				Coffee Consumption (cups/Day)				*p* for Trend	*p* for Interaction	*p* for Heterogeneity ^1^
SNP (Risk/Other)	Chr	Locus		0 to <0.5	0.5 to <1	1 to <3	≥3			
rs7756992(G/A)	6	CDKAL1							0.56	
GG+AG			Case/Total no.	424/2610	112/758	448/2957	165/1228			
			Pooled OR (CIs)	1.00 (reference)	0.92 (0.72–1.17)	0.93 (0.79–1.09)	0.83 (0.66–1.04)	0.11		0.96
AA			Case/Total no.	111/757	21/192	117/806	38/289			
			Pooled OR (CIs)	1.00 (reference)	0.79 (0.45–1.39)	1.03 (0.75–1.42)	0.99 (0.61–1.59)	0.87		0.70
rs10811661(T/C)	9	CDKN2A/B							0.97	
TT+CT			Case/Total no.	437/2697	113/776	452/3020	170/1228			
			Pooled OR (CIs)	1.00 (reference)	0.97 (0.75–1.23)	0.93 (0.79–1.09)	0.85 (0.68–1.06)	0.15		0.99
CC			Case/Total no.	97/670	20/174	113/743	34/290			
			Pooled OR (CIs)	1.00 (reference)	0.75 (0.44–1.31)	1.03 (0.74–1.43)	0.86 (0.52–1.41)	0.69		0.78
rs5215(C/T)	11	KCNJ11							0.73	
CC+CT			Case/Total no.	347/2183	77/605	371/2425	131/997			
			Pooled OR (CIs)	1.00 (reference)	0.83 (0.62–1.11)	0.98 (0.83–1.17)	0.87 (0.68–1.12)	0.34		0.61
TT			Case/Total no.	187/1179	55/343	194/1335	72/519			
			Pooled OR (CIs)	1.00 (reference)	1.08 (0.74–1.56)	0.90 (0.71–1.15)	0.80 (0.56–1.13)	0.22		0.42
rs163184(G/T)	11	KCNQ1							0.62	
GG+TG			Case/Total no.	381/2203	85/633	399/2428	133/1000			
			Pooled OR (CIs)	1.00 (reference)	0.81 (0.62–1.07)	0.99 (0.83–1.17)	0.81 (0.63–1.04)	0.15		0.34
TT			Case/Total no.	154/1164	48/314	166/1336	71/517			
			Pooled OR (CIs)	1.00 (reference)	1.10 (0.74–1.63)	0.90 (0.69–1.17)	0.94 (0.65–1.35)	0.78		0.20
rs3786897(A/G) *	19	PEPD							0.68	
AA+AG			Case/Total no.	403/2180	98/561	440/2453	149/909			
			Pooled OR (CIs)	1.00 (reference)	0.88 (0.68–1.14)	0.95 (0.80–1.12)	0.82 (0.65–1.04)	0.81		0.07
GG			Case/Total no.	87/543	19/123	87/538	34/200			
			Pooled OR (CIs)	1.00 (reference)	0.94 (0.52–1.70)	1.15 (0.79–1.65)	1.31 (0.78–2.18)	0.27		0.93

Abbreviations: ORs, odds ratios; CIs, Confidence intervals; SNP, single nucleotide polymorphisms; Chr, chromosome. MV adjusted: age (years, continuous), sex (men, women), BMI (<23 and ≥23 kg/m^2^), alcohol intake (never, ever for men; never, ever for women), smoking status (never, ever for HEXA, CAVAS, TWIN, never, pack-years < 10, 10–<20, 20–<30, 30–<40 ≥40 for men; never, pack-years < 5, 5–<10, ≥10 for women for KARE), education level (middle school or less, and high school or above), green tea intake (0–<2, and ≥2 cups/day), and total energy intake (kcal/day, continuous). ^1^ *p* for heterogeneity of odds ratios for top vs. bottom categories in MV adjusted model was presented. * Only the CAVAS and the KARE studies were included in analysis.

## Supplementary Materials

The following are available online at https://www.mdpi.com/1660-4601/17/15/5379/s1, Figure S1: Flow diagram of study population included in the analysis, Table S1: Subgroup analysis on the association of coffee consumption with type 2 diabetes stratified by type 2 diabetes-related SNPs, Table S2: Subgroup analysis on the association of coffee consumption with type 2 diabetes stratified by type 2 diabetes-related SNPs.
