# Supplementary material for: Coffee Consumption, Genetic Polymorphisms, and the Risk of Type 2 Diabetes Mellitus: A Pooled Analysis of Four Prospective Cohort Studies
Source: Int J Environ Res Public Health. 2020 Jul 26;17(15):5379. doi: 10.3390/ijerph17155379 (PMC7432682; doi:10.3390/ijerph17155379)
Supplement: Supplementary file 1 [file ijerph-17-05379-s001.pdf]

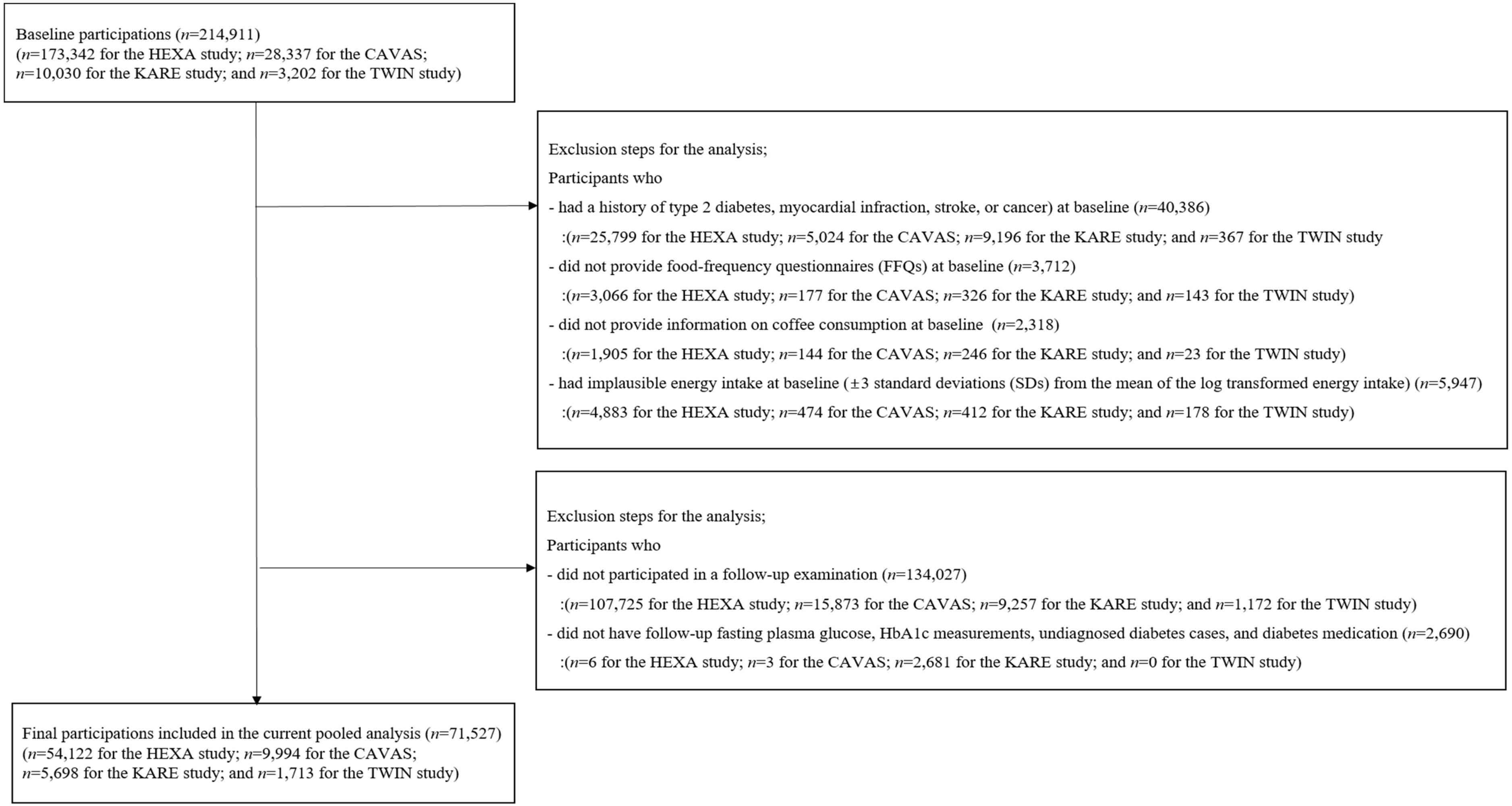

Figure S1. Flow diagram of study population included in the analysis.

Table S1. Subgroup analysis on the association of coffee consumption with type 2 diabetes stratified by type 2 diabetes-related SNPs.

SNP (Risk/Other)	Chr	Locus		Coffee Consumption (cups/day)				*p* for trend	*p* for interaction	*p* for heterogeneity[1)]
				**0 to <0.5**	**0.5 to <1**	**1 to <3**	**≥3**			
**rs7756992(G/A)**	6	CDKAL1							0.18	
GG			Case/Total no.	156/983	43/257	168/1,061	71/469			
			Pooled OR (CIs)	1.00 (reference)	1.16 (0.77-1.73)	1.00 (0.77-1.31)	1.04 (0.72-1.51)	0.79		0.53
AA+AG			Case/Total no.	379/2,384	90/693	397/2,702	132/1,048			
			Pooled OR (CIs)	1.00 (reference)	0.83 (0.63-1.09)	0.93 (0.78-1.10)	0.78 (0.61-1.00)	0.05		0.40
**rs10811661(T/C)**	9	CDKN2A/B								
TT			Case/Total no.	171/1,055	49/312	171/1,183	73/489		0.31	
			Pooled OR (CIs)	1.00 (reference)	1.12 (0.76-1.66)	0.87 (0.67-1.13)	0.99 (0.69-1.42)	0.96		0.66
CC+CT			Case/Total no.	363/2,312	84/638	394/2,580	131/1,029			
			Pooled OR (CIs)	1.00 (reference)	0.84 (0.64-1.11)	0.98 (0.83-1.17)	0.79 (0.62-1.02)	0.09		0.73
**rs5215(C/T)**	11	KCNJ11								
CC			Case/Total no.	94/517	20/128	102/565	33/224		0.60	
			Pooled OR (CIs)	1.00 (reference)	1.15 (0.62-2.11)	1.02 (0.71-1.47)	0.85 (0.49-1.45)	0.61		0.79
TT+CT			Case/Total no.	440/2,838	112/815	463/3,187	170/1,284			
			Pooled OR (CIs)	1.00 (reference)	0.90 (0.71-1.15)	0.95 (0.81-1.11)	0.86 (0.69-1.08)	0.21		0.97
**rs163184(G/T)**	11	KCNQ1							0.80	
GG			Case/Total no.	105/574	20/167	114/634	38/251			
			Pooled OR (CIs)	1.00 (reference)	0.76 (0.42-1.37)	1.09 (0.76-1.55)	0.81 (0.50-1.33)	0.55		0.71
TT+TG			Case/Total no.	430/2,793	113/780	451/3,130	166/1,266			
			Pooled OR (CIs)	1.00 (reference)	0.96 (0.75-1.22)	0.94 (0.81-1.10)	0.87 (0.70-1.09)	0.23		0.96
**rs3786897(A/G)***	19	PEPD							0.80	
AA			Case/Total no.	165/855	32/215	183/973	63/352			
			Pooled OR (CIs)	1.00 (reference)	0.74 (0.48-1.16)	0.91 (0.70-1.18)	0.89 (0.61-1.29)	0.63		0.46
GG+GA			Case/Total no.	325/1,868	85/469	344/2,018	120/757			
			Pooled OR (CIs)	1.00 (reference)	1.02 (0.76-1.35)	1.04 (0.86-1.25)	0.92 (0.71-1.20)	0.64		0.10

Abbreviations: ORs, Odds ratios; CIs, Confidence intervals; SNP, Single nucleotide polymorphisms; Chr, Chromosome. MV adjusted: age (years, continuous), sex (men, women), BMI (<23 and ≥23 kg/m^2), alcohol intake (never, ever for men; never, ever for women), smoking status (never, ever for HEXA, CAVAS, TWIN, never, pack-years <10, 10-<20, 20–<30, 30–<40 ≥40 for men; never, pack-years <5, 5–<10, ≥10 for women for KARE), education level (middle school or less, and high school or above), green tea intake (0-<2, and ≥2 cups/day), and total energy intake (kcal/day, continuous) [1)] *p* for heterogeneity of odds ratios for top vs bottom categories in MV adjusted model was presented * Only the CAVAS and the KARE studies were included in analysis.

Table S2. Subgroup analysis on the association of coffee consumption with type 2 diabetes stratified by type 2 diabetes-related SNPs.

SNP (Risk/Other)	Chr	Locus		coffee consumption (cups/day)				*p* for trend	*p* for interaction	*p* for heterogeneity[1)]
				0 to <0.5	0.5 to <1	1 to <3	≥3			
**rs7756992(G/A)**	6	CDKAL1							0.66	
GG			Case/Total no.	156/903	43/245	168/992	71/404			
			Pooled OR (CIs)	1.00 (reference)	1.16 (0.77-1.73)	1.00 (0.77-1.31)	1.04 (0.72-1.51)	0.80		0.40
AG			Case/Total no.	268/1,627	69/501	280/1,896	94/759			
			Pooled OR (CIs)	1.00 (reference)	0.83 (0.61-1.14)	0.89 (0.72-1.09)	0.74 (0.55-0.99)	0.02		0.14
AA			Case/Total no.	111/837	21/204	117/875	38/354			
			Pooled OR (CIs)	1.00 (reference)	0.79 (0.45-1.38)	1.03 (0.75-1.42)	0.98 (0.61-1.59)	0.96		0.70
**rs10811661(T/C)**	9	CDKN2A/B							0.46	
TT			Case/Total no.	171/716	49/219	171/815	73/302			
			Pooled OR (CIs)	1.00 (reference)	1.12 (0.76-1.66)	0.87 (0.67-1.13)	0.99 (0.69-1.42)	0.96		0.73
CT			Case/Total no.	266/1,195	64/341	281/1,319	97/536			
			Pooled OR (CIs)	1.00 (reference)	0.90 (0.65-1.24)	0.96 (0.78-1.18)	0.75 (0.56-1.00)	0.11		0.77
CC			Case/Total no.	97/589	20/187	113/685	34/266			
			Pooled OR (CIs)	1.00 (reference)	0.75 (0.43-1.31)	1.03 (0.74-1.43)	0.86 (0.52-1.41)	0.50		0.78
**rs5215(C/T)**	11	KCNJ11							0.73	
CC			Case/Total no.	94/517	20/128	102/565	33/224			
			Pooled OR (CIs)	1.00 (reference)	1.15 (0.62-2.11)	1.02 (0.71-1.47)	0.86 (0.50-1.47)	0.49		0.97
CT			Case/Total no.	253/1,613	57/438	269/1,775	98/734			
			Pooled OR (CIs)	1.00 (reference)	0.79 (0.56-1.09)	0.99 (0.81-1.22)	0.88 (0.66-1.18)	0.55		0.62
TT			Case/Total no.	187/1,225	55/377	194/1,412	72/550			
			Pooled OR (CIs)	1.00 (reference)	1.07 (0.73-1.54)	0.90 (0.70-1.15)	0.79 (0.56-1.12)	0.08		0.39
**rs163184(G/T)**	11	KCNQ1							0.66	

GG			Case/Total no.	105/574	20/167	114/634	38/251			
			Pooled OR (CIs)	1.00 (reference)	0.76 (0.42-1.37)	1.09 (0.76-1.55)	0.81 (0.50-1.33)	0.55		0.96
TG			Case/Total no.	276/1,629	65/466	285/1,794	95/749			
			Pooled OR (CIs)	1.00 (reference)	0.85 (0.62-1.16)	0.98 (0.80-1.20)	0.83 (0.62-1.12)	0.55		0.34
TT			Case/Total no.	154/1,164	48/314	166/1,336	71/517			
			Pooled OR (CIs)	1.00 (reference)	1.10 (0.74-1.62)	0.89 (0.69-1.16)	0.92 (0.64-1.33)	0.52		0.19
**rs3786897(A/G)***	19	PEPD							0.52	
AA			Case/Total no.	165/855	32/215	183/973	63/352			
			Pooled OR (CIs)	1.00 (reference)	0.74 (0.48-1.16)	0.91 (0.70-1.18)	0.89 (0.61-1.29)	0.63		0.46
GA			Case/Total no.	238/1,325	66/346	257/1,480	86/557			
			Pooled OR (CIs)	1.00 (reference)	1.01 (0.73-1.40)	0.98 (0.79-1.22)	0.79 (0.58-1.08)	0.26		0.10
GG			Case/Total no.	87/543	19/123	87/538	34/200			
			Pooled OR (CIs)	1.00 (reference)	0.94 (0.52-1.70)	1.15 (0.79-1.65)	1.31 (0.78-2.18)	0.27		0.30

Abbreviations: ORs, Odds ratios; CIs, Confidence intervals; SNP, Single nucleotide polymorphisms; Chr, Chromosome. MV adjusted: age (years, continuous), sex (men, women), BMI (<23 and ≥23 kg/m^2), alcohol intake (never, ever for men; never, ever for women), smoking status (never, ever for HEXA, CAVAS, TWIN, never, pack-years <10, 10–<20, 20–<30, 30-<40 ≥40 for men; never, pack-years <5, 5–<10, ≥10 for women for KARE), education level (middle school or less, and high school or above), green tea intake (0-<2, and ≥2 cups/day), and total energy intake (kcal/day, continuous)) [1)]*p* for heterogeneity of odds ratios for top vs bottom categories in MV adjusted model was presented * Only the CAVAS and the KARE studies were included in analysis.
